# Heparan Sulfate, Mucopolysaccharidosis IIIB and Sulfur Metabolism Disorders

**DOI:** 10.3390/antiox11040678

**Published:** 2022-03-30

**Authors:** Marta Kaczor-Kamińska, Kamil Kamiński, Maria Wróbel

**Affiliations:** 1 Chair of Medical Biochemistry, Faculty of Medicine, Jagiellonian University Medical College, 7 Kopernika St., 31-034 Krakow, Poland; mtk.wrobel@uj.edu.pl; 2 Department of Physical Chemistry, Faculty of Chemistry, Jagiellonian University, 2 Gronostajowa St., 30-387 Krakow, Poland; kaminski@chemia.uj.edu.pl

**Keywords:** glycosaminoglycans, heparin, Sanfilippo B syndrome, cysteine, sulfate, sulfane sulfur, sulfurtransferases, 3-mercaptopyruvate sulfurtransferase

## Abstract

Mucopolysaccharidosis, type IIIB (MPS IIIB) is a rare disease caused by mutations in the N-alpha-acetylglucosaminidase (*NAGLU*) gene resulting in decreased or absent enzyme activity. On the cellular level, the disorder is characterized by the massive lysosomal storage of heparan sulfate (HS)—one species of glycosaminoglycans. HS is a sulfur-rich macromolecule, and its accumulation should affect the turnover of total sulfur in cells; according to the studies presented here, it, indeed, does. The lysosomal degradation of HS in cells produces monosaccharides and inorganic sulfate (SO_4_^2−^). Sulfate is a product of L-cysteine metabolism, and any disruption of its levels affects the entire L-cysteine catabolism pathway, which was first reported in 2019. It is known that L-cysteine level is elevated in cells with the *Naglu*^−/−^ gene mutation and in selected tissues of individuals with MPS IIIB. The level of glutathione and the *Naglu*^−/−^ cells’ antioxidant potential are significantly reduced, as well as the activity of 3-mercaptopyruvate sulfurtransferase (MPST, EC 2.8.1.2) and the level of sulfane sulfur-containing compounds. The direct reason is not yet known. This paper attempts to identify some of cause-and-effect correlations that may lead to this condition and identifies research directions that should be explored.

## 1. Introduction

Rare diseases are disorders with a very low probability of occurrence in the population (the European Union Registration on Orphan Medicinal Products has estimated the rare disease prevalence as less than 1 person per 2000 [1]). They are usually genetic, chronic and severe, appearing mostly in childhood. Only a small group of people (about 5%) suffering from a rare disease are treated pharmacologically, which means that for the vast majority of them there is no effective therapy. One of these diseases is mucopolysaccharidosis, type IIIB (MPS IIIB) [OMIM 252920], which has an estimated incidence of 1/200,000 newborns [National Organization of Rare Disease]. Currently, there is no cure for MPS IIIB because there are no effective therapies which can stop or reverse it [2,3,4,5,6,7,8,9]. The disease itself is the result of a disruption of an ongoing process in the body of a healthy person. In this process, new molecules of polysaccharides made up of repeating disaccharide units—glycosaminoglycans (GAGs)—are continually produced, while older molecules are continually broken down. MPS IIIB is caused by a mutation in the N-alpha-acetylglucosaminidase (*NAGLU*) gene, resulting in the absence or decreased activity of the enzyme that is involved in the catabolism of GAGs. On the cellular level, the disorder is characterized by the massive lysosomal storage of heparan sulfate (HS)—a sulfur-rich macromolecule. Accumulation of such molecules should affect the turnover of total sulfur in the cells, and, as Kaczor-Kamińska and others showed [10,11], indeed, it does.

## 2. Glycosaminoglycans (GAGs)

GAGs (previously called mucopolysaccharides) accompany us from the beginning of fetal life [12,13,14], and this is true of all living organisms. From the chemical point of view, these are long, linear, unbranched heteropolysaccharides mostly bearing SO_3_^−^ groups [15]. This makes them biopolymers that are negatively charged and thus have good water solubility under physiological conditions. The standard for identifying individual GAGs is the presence of alternating repetitions of the corresponding disaccharide sequences in the macromolecules. At this point, liquid chromatography with MS detection is the only tool to effectively identify these compounds [16,17]. The GAGs known in the animal kingdom include [18,19] heparin, heparan sulfate (HS), chondroitin sulfate (CS), dermatan sulfate (DS), keratan sulfate (KS) and hyaluronic acid (HA). Analysis of the names of these compounds alone indicates that they are closely related to the tissues in which they occur in significant amounts (CS—from chondrocytes, DS—from skin/dermal tissue, KS from keratinocytes) or where they were first detected (heparin and HS from hepar—liver in Latin). An exception to the general rule in the case of the presence of sulfur in the GAGs molecules is HA, which does not have SO_3_^−^ groups and the negative charge is due to the presence of carboxyl groups [20].

Biosynthesis of GAGs is a very complex process involving many enzymes, and is still not completely understood [21]. GAGs are synthesized in cells of many tissues in which they play physiologically important functions. Universally, they are an important component of the intercellular matrix. In multicellular organisms this matrix is classically composed of fibrillar proteins such as collagen or elastin and mixtures of GAGs covalently linked to protein components [22,23]. There are organs in the body in which levels of these macromolecules are significantly higher, such as the eye [24] or heart muscle [25].

GAGs are macromolecules of which, compared to nucleic acids or proteins, biological significance has been underestimated until recently [26]. Classically they were perceived only as a non-protein component of proteoglycans in which the protein part is responsible for the biological activity of the whole molecule. This perception has changed considerably over the last decade and the concept of activity is no longer associated solely with proteins [26]. The participation of GAGs in so many biological processes, and their presence in almost all tissues, makes it reasonable to consider the option of using them as active substances in medicine [27]. The great importance and potential of GAGs in medicine may be evidenced by the fact that attempts have been made to realize ex vivo synthesis within the tools of classical organic synthesis of these compounds. Working with sugars is difficult due to the presence of multiple OH groups in the repeating unit, which require blocking and usually numerous steps [28] in order for them not to react. Nevertheless, the potential value of these compounds is so great for life sciences that attempts have been made to synthesize GAG fragments such as pentasaccharidic analogs of heparin [29] and longer structures [30,31]. Even simplified analogues of GAG are being developed, i.e., polymers with SO_3_^−^ containing groups as side chains, such as PAMPS [poly(2-acrylamido-2-methyl-1-propanesulfonic acid)]. These polymers exhibit some of the properties of GAGs, especially heparin, i.e., anticoagulant and anti-angiogenic properties [32].

Heparin is one of the most commonly used GAGs in medicine, among others, in the treatment of cardiovascular diseases and in some interventional cardiology procedures. The anticoagulant action of heparin results from its ability to bind with plasma protein antithrombin, which interacts with several serine proteases of the coagulation system, most importantly factors IIa (thrombin), Xa and IXa [33]. In terms of anticoagulant properties, the sequence of five sugar units is critical; therefore, similar properties to heparin are exhibited by its lower molecular mass analogues, low molecular weight heparin and synthetic pentasaccharide (Idraparinux). The pharmacokinetic properties of these systems are different, more desirable from the standpoint of chronic use, and their performance appears to be more predictable [34,35] to the point that they can be used by the patient without direct supervision by trained personnel. In addition to the commonly known anticoagulant properties of heparin, it (also low molecular weight heparin—LMWH) may also affect blood lipid balance [36].

Most of the physico-chemical and biological properties of GAGs are related to their structure or sequence (monosaccharide constituents and bonds between the disaccharide repeating units), conformation, chain flexibility, molecular weight and charge density [37,38]. Among these properties, the degree of GAGs sulfation (sulfation code), which is inherent in almost all GAGs, is crucial. Activities and functions of GAGs are mainly triggered by their interactions with a wide variety of biological molecules. Structural changes in GAGs that have no effect at the cellular level often have a huge impact at the organ/tissue or organism level, and changes in the GAG sulfation patterns are often associated with different disorders such as Alzheimer’s disease and cancer [38,39]. On the other hand, irregularities in normal physiological GAG degradation, leading to, e.g., endolysosomal HS accumulation, cause a group of less known rare diseases identified collectively as mucopolysaccharidoses (type III).

## 3. Heparan Sulfate (HS)

**Figure 1:**
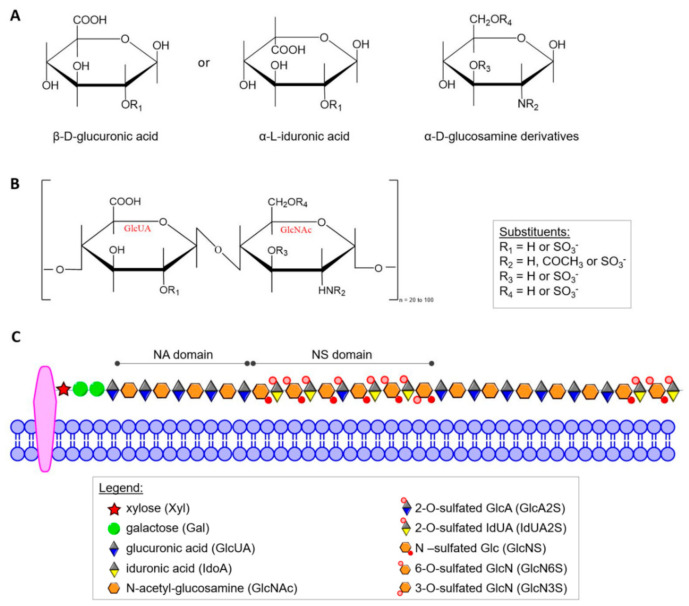
(**A**) Haworth projection of the heparin/HS monosaccharide units; (**B**) repeating GlcUA-GlcNAc disaccharide units of HS; (**C**) scheme of structural organization of the residues in HS. In the mature form of HS, two main types of domains can be distinguished: NA domains build mostly by non-modified N-acetyl-glucosamine moiety linked mainly to glucuronic acid residues, and NS domains that are composed by highly sulfated disaccharides units).

HS was defined as a distinct molecular entity by Jorpes and Gardell in 1948 [40] but elucidation of its structure has been a slow, painstaking process that is still underway. Today, it is known that HS is structurally closely related to heparin, and both differ from other GAGs in several important ways. α-d-Glucosamine (GlcN) and/or its derivatives and β-d-glucuronic acid (GlcUA) or its C5 epimer α-L-iduronic acid (IdUA) form the characteristic disaccharide repeat unit shown in Figure 1A,B. In contrast to most other GAGs, heparin and HS contain α-glycosidic linkages. In heparin almost all glucosamine residues (GlcN) contain sulfamide linkages, but a smaller number of GlcN are *N*-acetylated. Sulfate groups are linked by ester bonds to certain monosaccharides or by amide bonds to the amino group of GlcN. In addition to *N*-sulfate and *O*-sulfate on C6 of GlcN, heparin can also contain sulfate on C3 of the hexosamine and C2 of the uronic acid [41]. HS contains more *N*-acetyl groups, fewer *N*-sulfate groups, and a lower degree of *O*-sulfate groups. The sulfate content of heparin, although variable, approaches 2.7 sulfate per disaccharide unit in preparations with the highest biological activity [37], while HS contains only about one sulfate group per disaccharide. However, individual HS chains may have higher contents of this group and, additionally, often contain domains of extended sequences having low or high sulfation (Figure 1C) [42]. On account of structural heterogeneity, variability and polydisperse, HS cannot be considered a single compound but rather a family of related polymers [43]. Up to now, no rigorous formula of free HS has been established because within the carbohydrate chain of a given GAG there may be considerable variations of the constituent sugars, of the degree of sulfation and of the position of sulfate groups.

The structural features of HS chains, including length, uronic acid epimerization degree and sulfation patterns, play crucial roles in various biological processes [44]. In a given HS polysaccharide, negatively charged sulfate and carboxyl groups are arranged in various types of domains (Figure 1C) [43], but the domain organization of substituents along the HS chain and the composition of HS produced are distinctly regulated by different cells. The large HS structural diversity results from a tightly controlled biosynthetic pathway that is differently regulated in various organs, stages of development and pathologies, including cancer [45,46]. The current distinction between heparin and HS (and various GAGs in general) is not only based on carbohydrate structure [42,44,47], but also includes proteoglycan type and cellular distribution. Unlike other GAGs, heparin is an intracellular component of mast cells [43,48,49] and functions predominantly as an anticoagulant [32] and lipid-clearing agent [37]. HS may be extracellular or an integral and ubiquitous component of the cell surface in many tissues [43,50] including endothelium of blood vessels [51], amyloid [49,52], and brain [53]. HS on the cell surface provides cells with a mechanism to capture a wide range of extracellular effectors without requiring multiple new binding proteins [54].

**Figure 2:**
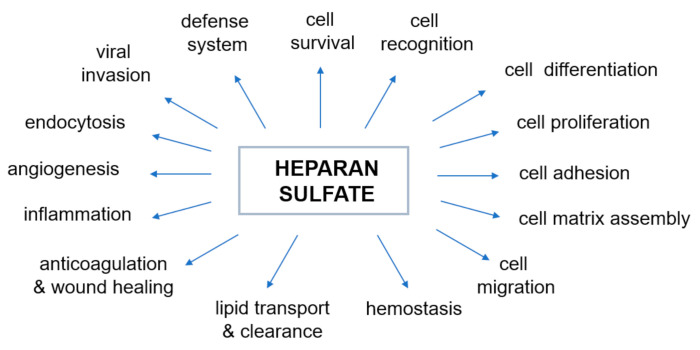
Biological activities modulated by the interaction of heparan sulfate with proteins. The diagram was prepared based on the information included in the following papers: [32,46,55,56,57,58,59,60,61,62].

The negative charge of HS largely determines the nature of interactions between proteoglycans and other molecules and ions of the extracellular matrix. The majority of physiological, as well as pathophysiological [45], activities of HS are due to interactions with various proteins. The most prominent type of interaction between HS and a protein is electrostatic. However, in some cases there is a significant contribution to the binding by non-electrostatic interaction such as hydrogen bonding and hydrophobic interactions [37]. The mechanism by which HS changes the activity of proteins through such relations has proved elusive. Over the last few decades, heparin and HS have been shown to bind and regulate the activities of many biologically important proteins, including enzymes, growth factors, extracellular matrix proteins, and the cell surface proteins of pathogens [54,55], and therefore play an essential role in various physiological and pathophysiological processes (Figure 2, prepared based on the information included in [32,46,55,56,57,58,59,60,61,62]). Genetic studies have shown that the properties of heparin and HS reflect their ability to regulate various biological process (Figure 2) such as, but not limited to, cell signaling and morphogenesis in vivo, growth regulation and tumor suppression [59,60,61,62]. It has also recently been discovered that excess of HS (and/or its derivatives) in specific cells and tissues affects sulfur metabolism disruption [10,11]. Therefore, our understanding of the nature of the heparin/HS-protein interactions at the molecular level offers a large number of potential therapeutic applications for these compounds and is necessary to understand normal physiological and pathophysiological processes.

## 4. Decomposition of Heparan Sulfate

**Figure 3:**
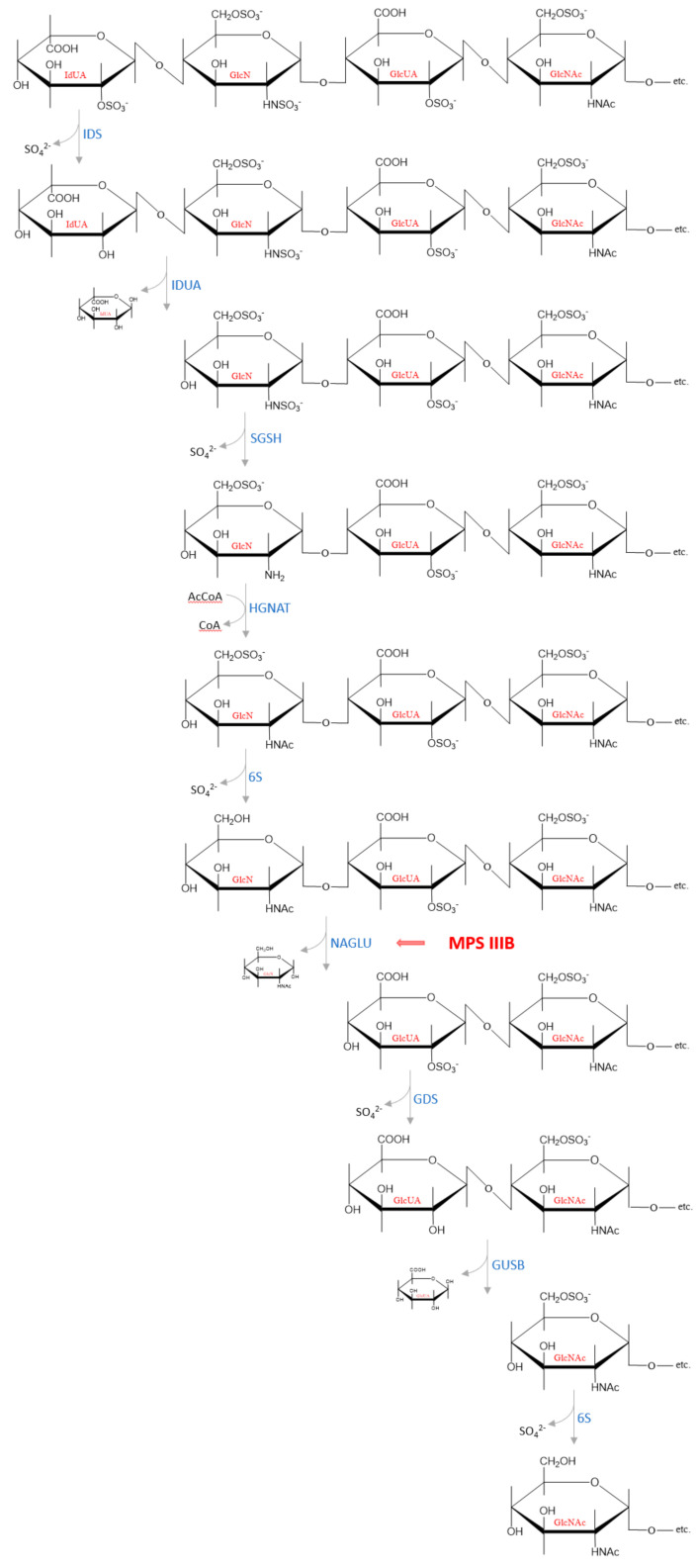
An example of how the subsequential degradation from the non-reducing end of the tetrasaccharide IdUA2S (α 1−4)—GlcNS6S (α 1−4)—GlcA2S (β 1−4)—GlcNAc6S occurs in the lysosome by HS degrading enzymes. Abbreviations: IdUA = iduronic acid; GlcN = glucosamine; GlcUA = glucuronic acid; GlcNAc = *N*−acetylglucosamine; IDS—iduronate−2−sulfatase (EC 3.1.6.13); IDUA—α−L−iduronidase (EC 3.2.1.76); SGSH—*N*−sulfoglucosamine sulfohydrolase (EC 3.10.1.1); HGNAT heparan−α−glucosaminide *N*−acetyltransferase (EC 2.3.1.78); 6S—*N*−acetylglucosamine−6−sulfatase (EC 3.1.6.14); NAGLU—α−*N*−acetylglucosaminidase (EC 3.2.1.50); GDS—glucuronate−2−sulfatase (EC 3.1.6.18); GUSB—β−glucuronidase (EC 3.2.1.21).

GAGs are present in cells in the form of proteoglycans, which are complex macromolecules composed of one or more GAG chains covalently attached to a protein core. Degradation of the protein moiety and the polysaccharide chains occurs independently of each other. This process begins with the proteolytic removal of the protein core of proteoglycans on the cell surface. Hydrolysis of some peptide bonds by proteases may take place extracellularly but the carbohydrate chain is degraded only within lysosomes [63,64]. The released GAG chains are then partially cleaved by enzymes such as endoglucuronidases or endohexosaminidases, which cut through the GAG macromolecules at several specific sites, resulting in formation of HS fragments and oligosaccharide fragments [65,66]. There are three lysosomal glycosidases that degrade HS oligosaccharides to monosaccharides: α-L-iduronidase (IDUA, EC 3.2.1.76) responsible for hydrolysis of unsulfated α-L-iduronosidic linkages; β-d-glucuronidase (GUSB, EC 3.2.1.31) that catalyzes hydrolysis of β-d-glucuronic acid from the non-reducing end of GAGs and α-*N*-acetylglucosaminidase (NAGLU, EC 3.2.1.50) that cleaves the *N*-acetyl-d-glucosamine α, 1 → 4 linkage between *N*-acetylglucosamine and the neighboring uronic acid from the non-reducing end in HS [64,66]. Complete degradation is achieved by the sequential action of endosulfatases, endodeacetylases and endoglycosidases at the non-reducing end of the chain [63,67]. An example of the subsequential cleavage of a HS chain by lysosomal enzymes is presented in Figure 3. The reaction products—monosaccharides and sulfate ions—are small enough to leave the lysosomal compartment. The degradation products are partially used in other metabolic processes, e.g., glucuronate and sulfate are used by the liver to conjugate with non-polar substances (e.g., some drugs such as diazepam, verapamil, and carbamazepine) or as final metabolites are excreted with the urine.

All HS-degrading enzymes in the lysosomes are exoactive, meaning they must act sequentially to fully breakdown the oligosaccharide chains. The HS chains derived from the same biosynthetic pool (tissue-, cells- and function-dependent) show similar distribution of NA-, NS-, as well as NA/NS- domains ratio (Figure 1C), and each domain type retains a typical substitution pattern. However, even though such a pattern is characterized by a defined GlcUA/IdUA moieties ratio and levels of different sulfate groups (linked by *N*-, 2-*O*-, 3-*O*, 6-*O*-), there is no evidence for the generation of predominant sequences of variously modified/substituted monosaccharide units during degradation process [43]. Therefore, depending on the initial HS sequence, there are many various structures of the oligosaccharide fragments that can accumulate in people suffering from MPS IIIB. However, the basic mechanisms of chain breakdown are thought to be similar in different organisms, but little is known about the actual structure of the entire decomposition process. A complete understanding of this enzymatic machinery responsible for HS degradation seems crucial for designing therapies for related diseases such as mucopolysaccharidoses.

## 5. Mucopolysaccharidosis, Type IIIB

Mucopolysaccharidoses are disorders caused by faulty degradation of sulfated mucopolysaccharides (sulfated GAGs), because of absent or decreased activity of an enzyme involved in this process (there is one exception—MPS type IX—where non-sulfated compound—HA is accumulated). The degradation process continues until a linkage occurs at the terminal end for which the deficient enzyme is specific. The genetic defect of the enzyme clearly indicates the stage at which the process of breaking down GAGs is stopped, as well as the subtype of MPS. Any further breakdowns are then stopped except for those brought about by endoglycosidases [63], such as NAGLU glycosidase. A deficiency, or mutations, in NAGLU has been linked to MPS IIIB (also known as Sanfilippo B syndrome) and is characterized by the lysosomal accumulation and urinary excretion of HS. The incidence of the mucopolysaccharidosis III is reported to be between 0.28 and 4.1 cases per 100,000 births, but its subtype B affects approximately 1 in 200,000 neonates (National Organization of Rare Disease).

**Figure 4:**
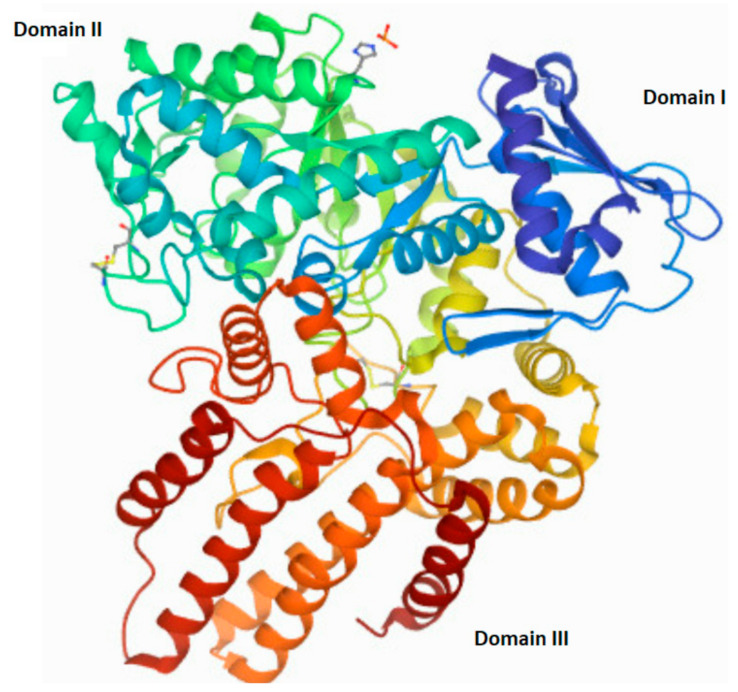
The tertiary structure of human *N*-acetyl-alpha-glucosaminidase (EC 3.2.1.50). A modified image from the RCSB PDB (rcsb.org) of PDB ID 4XWH_1 [68].

The human *NAGLU* gene is localized on chromosome 17q21.1 (gen length—10,212 bp) and consist of 7 exons (The National Center for Biotechnology Information, gene ID: 4669, accessed on 31 January 2022). In humans, alternative folding can result in five different transcript variants of *NAGLU* (four predicted and one established) that differ in the number of nucleotide pairs and lead to proteins differing in the number of amino acids. The one established *NALGU* cDNA (NM_000263.4) encodes a 743-amino-acid protein (NP_000254) that has six potential *N*-glycosylation sites. Using the NCBI database, as well as the National Institute of Environmental Health Sciences (NIH) website (https://manticore.niehs.nih.gov/snpinfo/snpinfo.html (accessed on 27 February 2022)), we checked whether there are changes (single nucleotide polymorphism—SNP) in the human *NAGLU* DNA that appear too often, to talk about the occurrence of a random mutation (differences in the population with a frequency above 1%). These analyses revealed, a significant number of single nucleotide polymorphisms present in the entire gene (3017 SNPs) as well as in its coding regions (cSNPs)—821 SNPs. Types of mutations found in the *NAGLU* coding region are as follows: 38 frame shift mutations, 285 synonymous mutations, 561 missense and 32 nonsense mutations, eight insertions and five deletions (according to the NIH database). Any non-synonymous mutation in the *NAGLU* gene may interfere with proper functioning, mostly by influence on expression and/or activity changes. Mutations located in the active site of the NAGLU enzymes, or those that result in the enzyme misfolding (causes their direction to proteasomal degradation instead of their cellular destination—lysosome), impair enzyme function. Mutations outside of the active site may alter the overall stability and/or function of the molecule [68]. Moreover, the number and/or location of the mutation correlates with the severity of the disease phenotype Clinical manifestations of MPS IIIB range from mild to severe and can be divided into three phases. In the first phase, there is a slight delay in the child’s development between 1 and 4 years of age. The second phase, which occurs around age 3–4, is characterized by hyperactivity, aggression, and a gradual loss of skills acquired during development, as well as a progressive intellectual decline. In the final stage, the child’s range of motion decreases significantly, even to the point where the patient loses mobility. Severe dementia and dysphagia occur [69,70,71,72,73,74]. Death usually occurs by the age of twenty, although there are some exceptions [75,76]. The structure of the native human NAGLU protein is shown in Figure 4.

The principal biochemical abnormality caused by the *NAGLU* gene or activity deficiency in MPS IIIB is the accumulation of lysosomal HS (the mechanism of HS storage has not yet been fully understood) and the elimination of this polysaccharide, or fragments derived from it, in body fluids [63]. Additionally, secondary storage products, such as: GM_2_ and GM_3_ gangliosides that play a role in central nervous systems pathology [63,77], inflammatory cytokines, reactive oxygen species [78] and globotriaosylsphingosine (LysoGb3) [79] have also been described. There are hypotheses concerning secondary storage product accumulation. Some have argued that this phenomenon may be due to the inhibition of relevant lysosomal enzymes by HS/GAGs, which when accumulating can selectively bind various hydrolases causing a decrease in their activity [79]. Others claim that they can cause critical disruption of the internal environment of the lysosome (for example, by changes in pH) and thus lead to reduced degradation of additional substrates [80].

However, many aspects of the MPS IIIB biochemistry are still unclear or even unknown. Therefore, understanding the roles of HS/GAGs role their effect on various biochemical processes in normal and pathogenic states seems to be very important, because no effective treatment for this disease is currently available [2], although a number of methods are being explored [3,4,5,6,7,8,9]. These methods include enzyme replacement therapies, substrate reduction therapies, gene therapy, hematopoietic stem cell transplantation and enzyme enhancement therapy [8,63]. Recently, Pearse and Iacovino [81] described clinical results of therapies for MPS IIIB such as genistein, BMN 250 and others [81]. Nevertheless, there is still a lack of approved MPS IIIB treatments, despite significant advances in research providing tools to understand their molecular basis, as well as legislation providing regulatory and economic incentives to accelerate the development of specific therapies.

## 6. GAGs as a Source of Sulfate

Lysosomal degradation of the carbohydrate portion of glycoproteins and GAGs produces monosaccharides and the greatest amount of inorganic sulfate (SO_4_^2−^) in cells compared to other sources such as catabolism of sulfur amino acids released during proteins breakdown and dietary supply [82,83,84]. Lysosomal inorganic sulfate can be reused in other biosynthetic pathways. Sulfates exit the lysosome either by secretion, i.e., through fusion of the lysosome with the cell membrane, or by entering the cytoplasm via sulfate transporters. This requires lysosomal acidic pH for optimal activity [82,85], which is maintained by the action of the lysosomal H^+^-ATPase. Some evidence suggests that SO_4_^2−^ transport is modulated by ATP independently from the lysosomal H^+^-ATPase action [82,86]. There are no data in the literature to indicate that sulfate is accumulated in cells and tissues, even though there is chronic exposition/ingestion of ‘above-normal’ sulfate levels [83]. Therefore, it can be assumed that inorganic sulfates are incorporated into several types of biomolecules, such as glycoproteins, glycosaminoglycans and glycolipids. Sulfate excreted by the kidneys derived mostly from oxidation of sulfur-containing amino acids [83].

**Figure 5:**
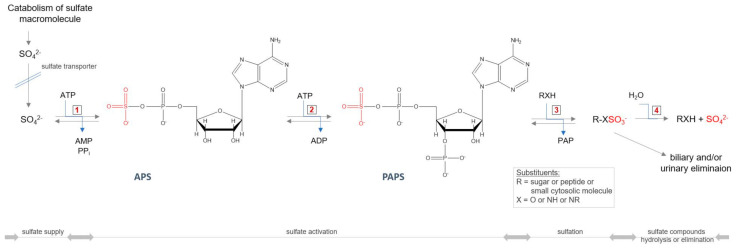
Cellular sulfate reuse pathway. Abbreviations: ATP—adenosine triphosphate; ADP—adenosine diphosphate; AMP—adenosine monophosphate; PP_i_—pyrophosphate; APS—adenosine−5′−phosphosulfate; PAPS—3′−phosphoadenosine−5′−phosphosulfate, PAP—3′−phosphoadenosine−5′−monophosphate; 1—ATP−sulfurylase (EC 2.7.7.4); 2—APS kinase (EC 2.7.1.25); 3—sulfotransferase (EC 2.8.2.-); 4—sulfatase (3.1.6.-).

All GAGs (except HA), during the synthesis process, require an additional modification step—sulfation—taking place in the Golgi apparatus. The incorporation of this group into the molecule requires the active sulfate donor compound 3’-phosphoadenosine-5’-phosphosulfate (PAPS) (Figure 5). The level of PAPS is tissue-dependent, and in humans is in the range 3.6–22.6 nmol/g tissue [87,88]. The intracellular PAPS availability may itself be under at least three multiple levels of control. The first control point is in the biosynthetic pathway of PAPS. The availability of PAPS for sulfation of GAGs significantly affects the rate of production of sulfated GAGs [89]. Thus, a defective conversion of 3′-adenosine phosphosulfate to PAPS, resulting in reduced levels of intracellular PAPS, leads to a synthesis of undersulfated proteoglycans in brachymorphic mice [90]. Another possible control level may be on the PAPS transport pathway to the Golgi apparatus, where sulfation occurs. A further factor that may limit the synthesis of PAPS is intracellular level of SO_4_^2−^—the third option. The Michaelis constant (K_m_) for SO_4_^2−^ at saturating MgATP in PAPS synthesis reaction is relatively high and equals 0.29 mM [91,92], and is on the order of magnitude of extracellular inorganic sulfate concentration, which is <100 µM [93] (the plasma concentration of inorganic sulfate is higher and maintained at levels 0.3–0.5 mM [94]). Thus, it is possible that PAPS synthesis is limited by the magnitude of the intracellular SO_4_^2−^ pool, and factors affecting the size of this pool may influence the PAPS levels and ultimately sulfation [90].

**Figure 6:**
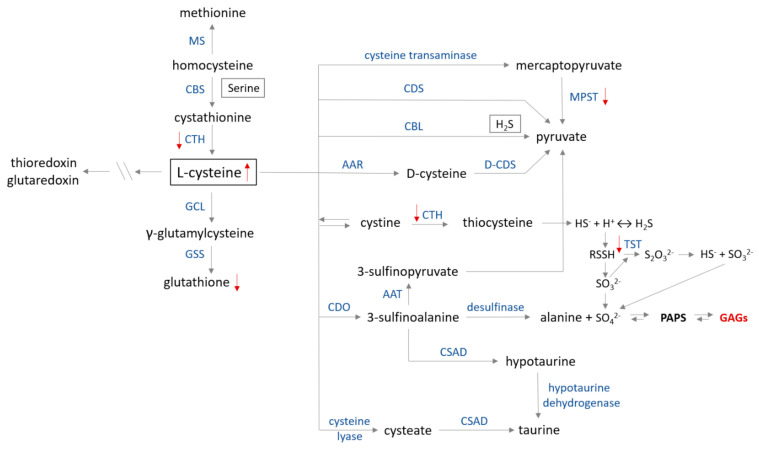
Cysteine metabolism map. The catabolism of cysteine involves seven pathways producing pyruvate, hydrogen sulfide, alanine or taurine. (1) In the first step, cysteine is converted to mercaptopyruvate in deamination reaction catalyzed by cysteine transaminase (CGT, EC 2.6.1.3). Subsequently, mercaptopyruvate is changed to pyruvate on the trans-sulfuration way by 3−mercaptopyruvate sulfurtransferase (MPST, EC 2.8.1.2)—the ‘mercaptopyruvate pathway’. Cysteine in reactions catalyzed by (2) cysteine desulfhydrase (CDS, EC 4.4.1.15) and (3) cystathionine β−lyase (CBL, EC 4.4.1.8) is directly converted to pyruvate and H_2_S (only CBL). (4) D−cysteine is formed from L−cysteine by the action of amino acid racemase (AAR, EC 5.1.1.10). (5) Cysteine is in dynamic equilibrium with cystine, which can be converted to thiocysteine in a reaction catalyzed by cystathionine γ−lyase (CTH, EC 4.4.1.1) and, in the following step, into H_2_S or persulfide. Conversion of persulfide into thiosulfate is catalyzed by thiosulfate sulfurtransferase (TST, EC 2.8.1.1) in the ‘thiosulfate cycle’. (6) Cysteine is transformed to 3−sulfinoalanine by cysteine dioxygenase (CDO, EC 1.13.11.20) via oxidation of a sulfhydryl group. Then, 3−sulfinoalanine can be converted to: (a) 3−sulfinopyruvate in a reaction catalyzed by aspartate transaminase (AAT, EC 2.6.1.1), which is further transformed to pyruvate; (b) to alanine by desulfinase (EC 4.1.1.12) in the ‘alanine pathway’; and (c) to taurine by sulfinoalanine decarboxylase (CSAD, EC 4.1.1.29) and hypotaurine dehydrogenase (EC 1.8.1.3) in the ‘taurine pathway’. (7) Cysteine can also be transformed to cysteate in reaction catalyzed by cysteine lyase (EC 4.4.1.10) and then to taurine by action of CSAD. Glutamate cystine ligase (GCL, EC 6.3.2.2) and glutathione synthase (GSS, EC 6.3.2.3) catalyze glutathione synthesis from cysteine. Further metabolism between methionine and cysteine is regulated by CTH, cystathionine β−synthase (CBS, EC 4.2.1.22) and methionine synthase (MS, EC 2.1.1.13). Red arrows show changes in sulfur metabolism caused by the GAGs accumulation (modified according to [97]).

The intracellular sulfate pool size depends on extracellular uptake through membrane transporters, catabolism of sulfur-containing amino acids and other thiols with the formation of sulfides that are then oxidized to sulfates, and degradation of sulfated molecules/macromolecules by sulfatases [95]. Although sulfate transporters are widely distributed in organs, various tissues may utilize specific compounds/groups of compounds as a major source of sulfate (e.g., lung cells utilize cysteine, kidney cells utilize cysteine and glutathione) [96]. All of the sulfate sources listed above work together to maintain the intracellular sulfate pool, but with different prevalence depending on the cell types [88]. Any disruption of cellular sulfate levels leads to a shift in the equilibrium state position of the reactions responsible for maintaining physiological levels of SO_4_^2−^, and indirectly, affects all reactions associated with these processes, as well as cellular sulfur metabolism in general (Figure 6). MPS IIIB is an example of a disorder in which there is an alteration in the amount of SO_4_^2−^ transferred from lysosomes to the cytoplasm after catabolism of HS macromolecules, as a result of some sulfate groups trapping in undegraded HS fragments deposited in lysosomes. It is known that undegraded GAGs are powerful inhibitors of GAGs sulfatases (K_i_ of HS is ≈50 nM) because the affinity of GAGs sulfatases for oligomers fragments of HS chain are usually higher than for the monomeric fragments [18].

However, there are no scientific data on intracellular sulfate levels determined in individuals with MPS IIIB. Based on the information we have, it could be argued that the overall pool of sulfate derived from GAGs degradation in lysosomes of cells with MPS IIIB may be lower compared to the same cell type but without the genetic defect causing the disease, because partially undegraded HS fragments are trapped in lysosomes. On the other hand, the total amount of sulfate could remain unchanged, due to existence of various sources of inorganic sulfate in cells. Any perturbation related to the SO_4_^2−^ levels can be compensated for to some extent. The idea that sulfur metabolism may be one of the mechanisms used by cells to maintain/restore constant sulfate levels is supported by the fact that in the selected tissues (liver, kidney, heart, spleen) of MPS IIIB individuals, a positive correlation between increased cellular GAGs levels and changes of some parameters characteristic for sulfur metabolism was observed (Figure 6, modified according to [97]) [10]. The third option, where the sulfate levels increase in cells with MPS IIIB, seems the least likely. Nevertheless, it cannot be completely ruled out because in a healthy cell there is a certain pool of compounds that need to be sulfated for the cell to function properly. Therefore, three different classes of sulfotransferases can be distinguished in the eukaryotic cell: lysosomal sulfotransferases, which participate in catabolism of sulfated macromolecules; cytosolic sulfotransferases, that are responsible for sulfation of small molecules such as hormones, amines, drugs and xenobiotics; and Golgi-associated sulfotransferases that sulfate larger compounds involved in the secretory pathway [88,98]. It seems that the fastest way to raise the sulfate concentration in the cell, when sulfate synthesis is deficient, is to transport SO_4_^2−^ from the extracellular space. Furthermore, cellular uptake of exogenous labeled HS by cells with MPS IIIB has been documented in the literature [11]. This is an interesting observation, showing that despite the accumulation of HS resulting from a mutation in the *NAGLU* gene, the cell takes up HS (and/or its derivatives) from the environment and localizes it both in the cytoplasm and in lysosomes [11]. Directing the absorbed HS into lysosomes is intended to completely degrade the molecule, resulting in the release of the SO_4_^2−^ groups it contains and replenishing the sulfate pool present in the cytoplasm. However, little is yet known about how sulfate levels affect total sulfur turnover in cells with MPS IIIB.

## 7. Effects of GAGs/HS Accumulation on Sulfur Metabolism

It was only in 2019 that information regarding changes in sulfur metabolism in MPS IIIB sufferers appeared for the first time [10,11]. As was demonstrated in both MPS IIIB mouse tissues (liver, spleen, heart, kidney) [10] and in the cellular model of MPS IIIB (the *Naglu*^−/−^ cells) [11], the cysteine level was elevated in the majority of studied cases (cysteine catabolism routes are presented in Figure 6). There may be many reasons for this condition because cysteine is a “metabolic hub” for biosynthesis of other S-containing small molecules (glutathione, coenzyme A and others) in cells; participates in many processes such as the synthesis of cysteinyl-tRNA required for protein synthesis, thiol-modified tRNA and is also a sulfur donor for the synthesis of Fe-S cluster cofactors or molybdenum cofactors [84,97]. However, it can be stated that, in general, the cysteine pool in the cells is increased under oxidizing conditions [99,100,101], but the research to-date does not demonstrate signs of oxidative stress in the *Naglu*^−/−^ cells [11] or in the majority of examined tissues [10]. In the *Naglu*^−/−^ cells, the ratio of reduced to oxidized glutathione concentration ([GSH]/[GSSG])—a major redox couple that determines the antioxidation capacity of the cells—is maintained, but the concentration values of both the reduced and oxidized forms of glutathione are significantly decreased (Figure 6) compared to the wild-type line [11]. Therefore, it may be assumed that cysteine (a substrate for the glutathione synthesis) is not used to synthesize glutathione molecules because the antioxidant potential of the *Naglu*^−/−^ cells was significantly reduced [11]. This naturally raises the question of what happens to the excess cysteine in the cells and what is the direct cause.

Based on the scientific data [97,102] it is known that excess of cysteine inhibits its metabolism by the transamination pathway (Figure 6). In normal conditions, cysteine overabundance is oxidized by cysteine dioxygenase and further transformed into taurine and sulfate, as well as is metabolized by desulfhydration pathways, resulting in hydrogen sulfide (H_2_S) excess and thiosulfate (S_2_O_3_^2−^) production (Figure 6) [97]. Unfortunately, no data are available for the parameters mentioned above measured in the population suffering from MPS. Therefore, based on the information we have [10,11], it can only be speculated that cysteine is diverted to the desulfuration pathway leading to the formation of compounds containing the so-called sulfane sulfur (a highly reactive, reduced sulfur atom at 0 or −1 oxidation state). However, preliminary research demonstrated [11] that the level of sulfane sulfur-containing compounds present in the *Naglu*^−/−^ cells was reduced as compared to the wild type cells. A similar observation was made in the liver, spleen and kidney of animals with MPS IIIB [10]. At the moment, these results are inconclusive, and it is not known whether the sulfane sulfur-containing compound levels in the MPS IIIB cells are reduced or whether there were simply fewer of these compounds there (which could also partly explain the low antioxidant potential of the GAGs-overloaded cells).

The next significant observation reported in both the MPS IIIB models by Kaczor-Kamińska and others [10,11] was a consistent reduction of the 3-mercaptopyruvate sulfurtransferase (MPST, EC 2.8.1.2, Figure 6) activity. It is known [101], that MPST activity is regulated by redox change. The activity is inhibited by oxidation resulting in the suppression of cysteine degradation, which may explain the observed elevated level of cysteine in the *Naglu*^−/−^ cells and tissues. MPST activity is regulated at the post-transcriptional level via redox-sensing molecular switches [101]. These switches can be classified into three subtypes: thioredoxin- glutathione-and glutaredoxin-specific, depending on reducing agents, demonstrating differences in their redox potential. If any data on these parameters were examined in patients with MPS IIIB, we might get closer to answering the question concerning possible causes of reduced MPST activity. Moreover, this could also provide information about redistribution of excess cysteine to other pathways, including the production of glutathione. Based on the available data, we know [11], that the level of glutathione in the *Naglu*^−/−^ cells is reduced. However, at the moment, it is unknown whether the reason for this is the inhibition/slowdown of glutathione production or the intensification of processes in the cell leading to the consumption of this low molecular weight antioxidant. MPST contains cysteine residues at the catalytic site [103]. Every single oxidative modification of the enzyme of this type changes or inhibits its activity; therefore, efficiently functioning cellular antioxidant defense systems are important. The formation of disulfide protects them against irreversible oxidation, as S-S bonds can be reduced by the thioredoxin/thioredoxin reductase (EC 1.8.1.9) system or the glutathione/glutharedoxin/glutathione reductase (EC 1.8.1.7) systems. Thus, MPST, as well as other two sulfurtransferases—thiosulfate sulfurtransferase (TST, also known as rhodanese, EC 2.8.1.1, Figure 6) and cystathionine γ-lyase (CTH, EC 4.4.1.1, Figure 6), partly contribute to maintaining the redox homeostasis and serves as an antioxidant protein [101,103,104,105,106]. Their action helps to maintain homeostasis of the sulfane sulfur-containing compounds levels in cells. Thus, expanded research is needed to explain why, despite the increased level of cysteine and decreased level of glutathione, there is a depletion of sulfurtransferases activity in the *Naglu*^−/−^ cells (MPST, TST, CTH) [11] and MPS IIIB tissues (MPST—kidney, spleen; TST—spleen and CTH—liver) [10].

It should also be mentioned that MPST and TST are regarded as sulfur-delivering enzymes for Fe-S cluster formation (the components of e.g., proteins mediating in electron transport processes). Therefore, their lower expression and/or activity (in the both tested MPS IIIB models, expression of MPST and TST remained at the control level, there was only one exception—the male *Naglu*^−/−^ kidney, where the MPST expression was significantly higher as compared to the control group [10,11]) can result in an increase of cysteine level, a decrease of sulfur available for regeneration/formation of Fe-S clusters and a decrease of ATP production/level. As previously mentioned, ATP is required for proper activity of PAPS (Figure 5). Thus, a decrease in ATP availability may result in inhibition/slowdown of PAPS formation causing a consequent reduction in sulfated compound levels in which synthesis is indirectly dependent on ATP, and an increase in the cellular sulfate level itself. As demonstrated, little is known about the GAGs-induced changes affecting sulfur metabolism in individuals with MPS IIIB (with MPS in general).

## 8. Perspectives

This paper aims to draw the attention of the scientific community to a research problem that has not been sufficiently explored to date. To this purpose, we collected available data on documented changes occurring in sulfur metabolism in MPS IIIB patients and attempted to identify some causal correlations and indicate directions that should be explored. The observations presented in the paper are new and can significantly contribute to the expansion of knowledge related to MPS IIIB. They may point to new directions of research or therapeutic approaches, which, we hope, in the future will, if not cure, improve the comfort of patients affected by this disease.
